# From kraepelin to the present. Dementia praecox – a case study

**DOI:** 10.1192/j.eurpsy.2021.2105

**Published:** 2021-08-13

**Authors:** M.D.R. Basto, L. Lopes, A. Miguel, A. Certo, Â. Venâncio, A. Horta

**Affiliations:** Serviço De Psiquiatra E Saúde Mental, Centro Hospitalar de Vila Nova de Gaia e Espinho, Vila Nova de Gaia, Portugal

**Keywords:** History, psychosis, Dementia praecox, Kraepelin

## Abstract

**Introduction:**

In the late XIX^th^ century Kraepelin described a new nosologic division for the psycothic disorders – Paranoia and Dementia Praecox. He emphasized that dementia praecox is a central nervous system disease, involving permanent lesions on cerebral cortex. Besides biological deterioration, it appears as the result of psychic degenerative process. From the mid-20th century onward, antipsychotic drugs had been robustly generalized, and in parallel to the current classifications, residual symptoms in schizophrenia tend to be rare but still prevail in our patients.

**Objectives:**

The aim of our work is to report a clinical case of residual schizophrenia in parallel with the classic classification of Dementia Praecox and also do an overview of this disorder and its historical perspective.

**Methods:**

We conducted clinical interviews with the patient and family members, reviewed clinical records and conducted a query in the MEDLINE database using the terms &quot; Dementia Praecox &quot;, “Psychosis”, “Paranoia”, “Kraepelin”, “History”.

**Results:**

We present the clinical case of a 74-year-old man with onset of psychotic symptoms on his twenties and diagnosed with Schizophrenia. In the past years, after acute psychotic episodes it was increasingly difficult to return to prior levels of functioning. Currently, he was brought to psychiatric emergency ward presenting bizarre behavior, stereotyped movements and speech disturbances, which reveal disorganized thinking and inability to express his emotions.

**Conclusions:**

Although these syndromes are nowadays relatively rare, it is important to keep them in mind, in order to understand the natural progression of psychotic diseases, improve their rehabilitative treatment and prognosis.

**Disclosure:**

No significant relationships.

